# Risk factors for heart failure hospitalizations among patients with atrial fibrillation

**DOI:** 10.1371/journal.pone.0191736

**Published:** 2018-02-02

**Authors:** Lucien Eggimann, Steffen Blum, Stefanie Aeschbacher, Andreas Reusser, Peter Ammann, Paul Erne, Giorgio Moschovitis, Marcello Di Valentino, Dipen Shah, Jürg Schläpfer, Nadine Mondet, Michael Kühne, Christian Sticherling, Stefan Osswald, David Conen

**Affiliations:** 1 Division of Cardiology, Department of Medicine, University Hospital Basel, University of Basel, Basel, Switzerland; 2 Cardiovascular Research Institute Basel, University Hospital Basel, University of Basel, Basel, Switzerland; 3 Division of Internal Medicine, Department of Medicine, University Hospital Basel, University of Basel, Basel, Switzerland; 4 Division of Cardiology, Kantonsspital St. Gallen, St. Gallen, Switzerland; 5 Laboratory for Signal Transduction, Department of Biomedicine, University Hospital Basel, Basel, Switzerland; 6 Division of Cardiology, Ospedale Regionale di Lugano, Ticino, Switzerland; 7 Division of Cardiology, Ospedale San Giovanni Bellinzona, Ticino, Switzerland; 8 Division of Cardiology, University Hospital Geneva, Geneva, Switzerland; 9 Service of Cardiology, University Hospital Lausanne, Lausanne, Switzerland; 10 Population Health Research Institute, McMaster University, Hamilton, Ontario, Canada; Ospedale del Cuore G Pasquinucci Fondazione Toscana Gabriele Monasterio di Massa, ITALY

## Abstract

**Background:**

Patients with atrial fibrillation (AF) have an increased risk for the development of heart failure (HF). In this study, we aimed to detect predictors of HF hospitalizations in an unselected AF population.

**Methods:**

The Basel Atrial Fibrillation Cohort Study is an ongoing observational multicenter cohort study in Switzerland. For this analysis, 1193 patients with documented AF underwent clinical examination, venous blood sampling and resting 12-lead ECG at baseline. Questionnaires about lifestyle and medical history were obtained in person at baseline and during yearly follow-up phone calls. HF hospitalizations were validated by two independent physicians. Cox regression analyses were performed using a forward selection strategy.

**Results:**

Overall, 29.8% of all patients were female and mean age was 69 ±12 years. Mean follow-up time was 3.7 ±1.5 years. Hospitalization for HF occurred in 110 patients, corresponding to an incidence of 2.5 events per 100 person years of follow-up. Independent predictors for HF were body mass index (HR 1.40 [95%CI 1.17; 1.66], p = 0.0002), chronic kidney disease (2.27 [1.49; 3.45], p = 0.0001), diabetes mellitus (2.13 [1.41; 3.24], p = 0.0004), QTc interval (1.25 [1.04; 1.49], p = 0.02), brain natriuretic peptide (2.19 [1.73; 2.77], p<0.0001), diastolic blood pressure (0.79 [0.65; 0.96], p = 0.02), history of pulmonary vein isolation or electrical cardioversion (0.54 [0.36; 0.80], p = 0.003) and serum chloride (0.82 [0.70; 0.96], p = 0.02).

**Conclusions:**

In this unselected AF population, several traditional cardiovascular risk factors and arrhythmia interventions predicted HF hospitalizations, providing potential opportunities for the implementation of strategies to reduce HF among AF patients.

## Introduction

Atrial Fibrillation (AF) is the most common cardiac arrhythmia [1] and its incidence is expected to further increase with the ageing of the population [2]. Lifetime risk for AF development is 1 in 4 among individuals aged 40 years or older [3], Patients with AF face an increased risk of developing stroke, heart failure (HF), cognitive dysfunction and death [4–7].

Prior studies have highlighted that HF is an important comorbidity associated with AF, and both entities frequently coexist [6]. AF occurs in more than half of individuals with HF and HF occurs in more than one third of individuals with AF [8]. While many studies have targeted the prevention of stroke in AF patients [9], little attention has been placed on the prevention of HF, despite its high prevalence and its important impact on affected patients [6, 10–12]. There is also an important economical impact, as HF predicts future hospitalizations in AF patients [13].

Therefore, it is important to recognize risk factors for incident HF events among AF patients, potentially providing opportunities for preventive measures. However, comprehensive assessments on risk stratification for HF in patients with AF are scarce. To our knowledge there has no study been assessing the role of biomarkers such as brain natriuretic peptide (BNP) to predict HF in AF patients [11, 14–19]. Furthermore, prior studies enrolled either patients without history of HF or did not differentiate between known HF and no history of HF [11, 14–19].

Therefore, this study aimed to assess different predictors for HF hospitalizations including medical history-, lifestyle-, ECG- and biomarker data in an unselected cohort of patients with established AF.

## Methods

### Study sample

The Basel Atrial Fibrillation Cohort Study (BEAT- AF) is an ongoing prospective, observational multicenter cohort study performed in 7 centers in Switzerland. Overall, 1553 patients with documented AF were included between 2010 and 2014. The main inclusion criterion for study participation was previously documented AF. Patients with exclusively short, temporary AF episodes (e.g. AF following cardiac surgery) were excluded. Patients suffering from an acute disease were enrolled after they had recovered from their acute illness. Of 1553 patients enrolled in this study, 324 patients were excluded from this analysis because of missing laboratory values and 36 patients due to missing ECG-, medical history- or lifestyle data, resulting in a total number of 1193 patients. A total of 86 (7.2%) patients were lost to follow-up. The study protocol was approved by the local ethics committees (Ethikkommission Nordwest- und Zentralschweiz) and informed written consent was obtained from every participating patient.

### Baseline assessments

All study patients were asked to complete a questionnaire about lifestyle, personal factors and comorbidities. Smoking status was categorized in current smokers and non-current smokers (past or never smokers). Exercise was graded in either doing any regular exercise or no regular exercise. Daily alcohol intake was classified as no alcohol consumption, moderate alcohol consumption (men > 0 < 24 g/d, women > 0 < 12 g/d) and high alcohol consumption (men > 24 g/d, women >12 g/d) [20]. Current medication, medical history, comorbidities and history of arrhythmia interventions (defined as previous pulmonary vein isolation or electrical cardioversion) were recorded.

At baseline, all patients underwent clinical examination, resting 12- lead ECG recording and venous blood sampling at the local study center. Standard blood and chemistry results were obtained at the local study center. Height and weight were directly measured using standardized devices. Body mass index (BMI) was calculated as weight in kg divided by height in meters squared. Blood pressure was measured twice in a resting position. Resting heart rate was obtained from the resting ECG. A 12-lead ECG was obtained with a standard ECG device in every center. QT interval was adjusted for mean heart rate (QTc) using the Bazett formula [21]. Estimated glomerular filtration rate (eGFR) was calculated using the Chronic Kidney Disease Epidemiology Collaboration (CKD-EPI) equation [22]. Antiarrhythmic drug (AAD) intake was defined as treatment with rhythm control medication. AF was classified according to the guidelines of the European Society of Cardiology into paroxysmal AF (self-terminating, usually within 48 hours), persistent AF (episodes lasting longer than 7 days or requiring termination by cardioversion) or permanent AF (AF is accepted by the patient and the physician and no attempts to restore sinus rhythm are performed) [1].

### Follow- up assessments

A yearly follow- up by mail and phone call was performed to update current AF type, smoking status, exercise habits and detailed medical history. The main outcome measure for this analysis was hospitalization for HF. Hospitalization for HF was defined as at least overnight admission to the hospital with clinical symptoms (e.g. leg swelling/leg edema, distension of neck veins, positive hepato-jugular reflux, rales or 3rd heart sound) and signs of HF resulting from an abnormality of cardiac structure or function. For this study we used all outcome data available until 7^th^ of April 2017. All HF events were validated by two independent physicians in a standardized procedure, using all available information. In case of discordance, a third physician was involved.

### Statistical analysis

Baseline characteristics for all patients were stratified by presence of known HF at baseline. Categorical variables were presented as counts (percentages) and compared using Chi-square tests. Distribution of continuous variables was checked using skewness, kurtosis and visual inspection of the histogram. Normally distributed continuous variables were presented as mean ± standard deviation (SD) and compared using Student’s t-tests. Skewed variables were presented as median (interquartile range) and compared using Wilcoxon rank sum tests. Person-years of follow-up were calculated as the time between the date of study enrollment and the first occurrence of hospitalization for HF, death, loss to follow-up or 7^th^ of April 2017.

To identify predictors for HF hospitalizations, we constructed Cox regression models to assess hazard ratios (HR) and 95% confidence intervals (95%CI) and to adjust for potential confounders. In a first step, continuous variables were used as quartiles in order to check for linearity of the association. If the shape of the association was linear, these variables were used in the continuous form. For continuous variables, HR were calculated per one SD increase. We then used a forward selection process to obtain a set of independent predictors. A p-value of 0.05 was used as cut-off to enter the model. Variables used for the forward selection were: Sex, age, BMI, resting heart frequency, systolic blood pressure, diastolic blood pressure, QRS interval, QTc interval, alcohol consumption (moderate and high alcohol consumption), known history of HF at baseline as well as the laboratory parameters albumin, brain natriuretic peptide (BNP), C-reactive protein, chloride, potassium, serum lactate dehydrogenase, sodium and eGFR. History of stroke, AF type (paroxysmal/ non paroxysmal), diabetes mellitus, device implantation, atrial flutter, chronic kidney disease, obstructive sleep apnea, cardiac valve surgery, coronary artery disease (CAD), hypertension, exercise, smoking habits, beta blocker intake, AAD intake, and history of arrhythmia intervention were included as time updated variables in the Cox regression model.

Given the strong impact of a previous history of HF on future HF hospitalizations [23, 24], we performed a sensitivity analysis among those patients without a history of HF (n = 951). The proportional hazard assumption was checked by including a logarithm of time x predictor variable into the models, and no violations were detected [25]. As a sensitivity analysis we recalculated the main models using competing risk subdistribution hazard models as described by Fine and Gray [26]. A two-sided p value <0.05 was considered to indicate statistical significance. All statistical analyses were performed using SAS 9.4 (SAS Corporation, Cary, North Carolina, USA).

## Results

We included 1193 patients in this study, 951 (79.7%) of them without a previous history of HF at baseline. Baseline characteristics stratified by history of HF at baseline are presented in Table 1. Mean age of all patients was 69 ±12 years. Overall, 29.8% were females and mean BMI was 27 ±4.6 kg/m^2^, both without significant difference in patients with and without HF at baseline. Paroxysmal AF was present in 32.6% and 60.4% of the patients with and without HF (p<0.0001). Individuals with HF at baseline had more often diabetes mellitus (p = 0.0008). Median BNP levels were 243 (128; 458) ng/L and 109 (52; 209) ng/L for patients with and without a baseline history of HF (p<0.0001).

**Table 1:** Baseline characteristics.

	All patients (n = 1193)	Patients without known HF at baseline (n = 951)	Patients with known HF at baseline (n = 242)	p-value*
Age (years)	68.9 (±11.7)	68.1 (±11.8)	72 (±10.5)	<0.0001
Female sex	356 (29.8%)	290 (30.5%)	66 (27.3%)	0.33
BMI (kg/m^2^)	27 (±4.6)	26.9 (±4.4)	27.4 (±5.1)	0.15
SBP (mmHg)	134.9 (±18.8)	136.7 (±18.4)	127.8 (±19)	<0.0001
DBP (mmHg)	78.4 (±12.3)	79.3 (± 11.9)	74.7 (±13.2)	<0.0001
Heart frequency (bpm)	67 (58; 80)	66 (57; 79)	70 (60; 83)	0.003
Paroxysmal AF	653 (54.7%)	574 (60.4%)	79 (32.6%)	<0.0001
Diabetes mellitus	171 (14.3%)	120 (12.6%)	51 (21.1%)	0.0008
Hypertension	825 (69.2%)	630 (66.3%)	195 (80.6%)	<0.0001
History of stroke	155 (13%)	121 (12.7%)	34 (14.1%)	0.58
Obstructive sleep apnea	135 (11.3%)	96 (10.1%)	39 (16.1%)	0.008
CAD	247 (20.7%)	162 (17%)	85 (35.1%)	<0.0001
History of valve surgery	91 (7.6%)	51 (5.4%)	40 (16.5%)	<0.0001
Chronic kidney disease	193 (16.2%)	96 (10.1%)	97 (40.1%)	<0.0001
History of arrhythmia intervention	544 (45.6%)	423 (44.5%)	121 (50%)	0.12
Current smoker	107 (9%)	82 (8.6%)	25 (10.3%)	0.41
Regular exercise	636 (53.3%)	539 (56.7%)	97 (40.1%)	<0.0001
Beta blocker intake	832 (69.7%)	636 (66.9%)	196 (81%)	<0.0001
AAD intake	340 (28.5%)	266 (28%)	74 (30.6%)	0.42
QTc interval (ms)	438.1 (±43.6)	433.4 (± 42.5)	456.4 (±43.1)	<0.0001
BNP (ng/L)	128 (58; 252)	109 (52; 209)	243 (128; 458)	<0.0001
Chloride (mmol/l)	102.5 (±3.15)	102.8 (±3)	101.5 (±3.6)	<0.0001

Data are presented as number (percentages) or mean (± standard deviation) or median (interquartile range), as appropriate. BMI = body mass index; SBP = systolic blood pressure; DBP = diastolic blood pressure; CAD = coronary heart disease; AAD = antiarrhythmic drug; BNP = brain natriuretic peptide; History of arrhythmia intervention = previous pulmonary vein ablation or/and electrical cardioversion. Heart frequency and BNP were log-transformed.

*P-values compare patients with and patients without known HF at baseline and are based on chi-square tests or t-tests/Wilcoxon rank sum tests, as appropriate.

Over a mean follow-up of 3.7±1.5 years, 110 patients were hospitalized for HF. The incidence for HF hospitalizations was 2.5 events per 100 person years of follow-up. Multivariable adjusted predictors for incident HF are presented in Table 2, and include BMI (HR 1.40 [95%CI 1.17; 1.66], p = 0.0002), chronic kidney disease (2.27 [1.49; 3.45], p = 0.0001), diabetes mellitus (2.13 [1.41; 3.24], p = 0.0004), QTc interval (1.25 [1.04; 1.49], p = 0.018), BNP (2.19 [1.73; 2.77], p<0.0001), diastolic blood pressure (0.79 [0.65; 0.96], p = 0.016), history of arrhythmia intervention (0.54 [0.36; 0.80], p = 0.003) and serum chloride (0.82 [0.70; 0.96], p = 0.015).

**Table 2:** Predictors of hospitalization for heart failure (HF) in patients with and without known HF at baseline.

	HR (95% CI)	p-value
**Predictor in patients with and without known HF at baseline**	**n = 1193**	
BMI (kg/m^2^)	1.40 (1.17; 1.66)	0.0002
Diastolic blood pressure (mmHg)	0.79 (0.65; 0.96)	0.016
Chronic kidney disease	2.27 (1.49; 3.45)	0.0001
Diabetes mellitus	2.13 (1.41; 3.24)	0.0004
History of arrhythmia intervention	0.54 (0.36; 0.80)	0.003
QTc interval (ms)	1.25 (1.04; 1.49)	0.018
BNP (ng/L)	2.19 (1.73; 2.77)	< 0.0001
Chloride (mmol/l)	0.82 (0.70; 0.96)	0.015
**Predictor in patients without known HF at baseline**	**n = 951**	
Age (years)	1.51 (1.002; 2.29)	0.049
BMI (kg/m^2^)	1.46 (1.14; 1.87)	0.003
Diabetes mellitus	2.72 (1.57; 4.71)	0.0004
History of valve surgery	3.10 (1.58; 6.10)	0.001
History of arrhythmia intervention	0.41 (0.23; 0.76)	0.004
QTc interval (ms)	1.44 (1.14; 1.83)	0.002
BNP (ng/L)	1.92 (1.30; 2.83)	0.001

Data are Hazard Ratios (HR) and 95% confidence intervals (95%CI). HR for continuous variables are per one standard deviation increase. BMI = body mass index; BNP = brain natriuretic peptide; history of arrhythmia intervention = previous pulmonary vein ablation or/and electrical cardioversion. BNP was log-transformed.

Among patients without known HF at baseline, mean follow-up was 3.9 (±1.4) years. Hospitalization for HF occurred in 60 patients. The incidence rate was 1.6 per 100 person years of follow-up. Multivariable adjusted predictors associated with HF hospitalizations were age (HR 1.51 [95%CI 1.002; 2.29], p = 0.049), BMI (1.46 [1.14; 1.87], p = 0.003), diabetes mellitus (2.72 [1.57; 4.71], p = 0.0004), history of valve surgery (3.10 [1.58; 6.10], p = 0.001), QTc interval on the ECG (1.44 [1.14; 1.83], p = 0.002), BNP (1.92 [1.30; 2.83], p = 0.001) and history of arrhythmia intervention (0.41 [0.23; 0.76], p = 0.004), as shown in Table 2.

Results of the sensitivity analyses are presented in S1 Table. By taking death as a competing risk into account, diastolic blood pressure and QTc interval were no longer significant predictors of HF hospitalizations in the whole population. The HR for all other predictors remained similar compared to the main model.

## Discussion

In this prospective cohort study of patients with AF, several independent risk factors for HF hospitalizations were identified. Higher BMI, elevated BNP, diabetes mellitus, chronic kidney disease and longer QTc interval were associated with an increased risk of HF, whereas patients with higher diastolic blood pressure, higher serum chloride and history of arrhythmia-related interventions were significantly less likely to develop HF. Finally, most risk factors identified in this study were similar whether patients had known HF at baseline or not. Thus, although the absolute risk for HF development is higher among patients with known HF [16, 18], preventive strategies are probably the same.

Several of these predictors are established cardiovascular risk factors and amenable to prevention, providing a great potential opportunity to reduce the HF burden among patients with AF. Both obesity and type 2 diabetes mellitus have been firmly related to the occurrence of cardiovascular events [27–29], and both were related to AF and HF incidence [29–32]. Moreover, prior studies have shown that they were associated with incident HF in patients with AF [11, 15, 17], a finding that is confirmed by our results. On the other hand, some studies suggested that a higher BMI is associated with a lower all-cause mortality among AF patients [33, 34]. Additionally, in a cohort of patients suffering from AF and HF together, overweight and obesity were also associated with a lower risk of all-cause mortality [35]. Whether these differential findings suggests different associations between BMI and outcomes or whether they are at least in part due to residual confounding should be assessed in future studies.

A prolonged QTc interval has been associated with a higher rate of cardiovascular events including HF and death in a population based cohort [36]. However, in patients with AF there is only little information available about this association. The results of our study presenting prolonged QTc interval as an independent predictor for HF should be further investigated. QTc interval can be easily assessed in clinical daily routine and therefore, it may be a simple tool for risk stratification.

BNP levels are of major importance for the management of patients with acute or chronic HF [37, 38]. To our knowledge, there is no prospective study assessing the relationship between BNP levels and incident HF in patients with AF. The findings of our study indicate that BNP is a strong predictor of HF also in patients with AF, who usually have a higher burden of comorbidities and higher BNP levels compared to the general population [39, 40]. As BNP levels were also predictive of incident HF among patients without a history of HF, BNP may help to identify AF patients at increased risk of HF in the future. Previous history of known HF, which was associated with increased risk of recurring HF generally [23, 24] and in AF patients [16, 18], was not a predictor for future HF hospitalizations in our study, which might be a result of the correlation between BNP and previous history of HF.

Several studies showed the prognostic implications of renal function in patients with heart failure [41] and the relationship between renal function and cardiovascular morbidity and mortality [42]. Additionally, renal dysfunction was also reported to promote HF in patients with AF [14, 17], which is in line with our results. Low serum chloride was a predictor for HF in our study, similar to a previously published population based cohort [43] and among patients with hypertension [44]. Moreover, there are some prognostic data available in patients with acute decompensated [45] and chronic heart failure [46].

Higher DBP was associated with a better outcome in the current study. Previous studies showed that low DBP was an independent predictor of re-hospitalization for HF in a cohort of patients with acute decompensated HF [47]. Another study also related low DBP to higher mortality in patients with systolic HF [48]. Low DBP might impair coronary blood flow during diastole, which might result in a decreased coronary perfusion that affects the myocardium and promotes the development of HF. Additionally, with increasing aortic stiffness, diastolic BP is decreasing and the prognosis for adverse outcomes is increasing. Finally, arrhythmia-related interventions but not AAD intake was associated with a reduced risk of incident HF in our study. Prior studies on rhythm versus rate control did not show an advantage of rhythm control [49]. Whether the current findings are due to selection bias or whether more modern rhythm control interventions such as pulmonary vein isolation do have a prognostic benefit is a question of ongoing trials.

Several risk scores to predict HF among AF patients have been evaluated. Imai et al. included age, heart rate, hypertension und history of HF in their score [18]. In Suzuki et al., HF, anemia, renal dysfunction, diabetes mellitus and the use of diuretics were included [17]. More studies are needed to assess whether these differences across studies are due to differences in the patient populations, ethnicity, methodology used or other factors.

The strengths of this study are the large unselected cohort of extensively phenotyped patients who were followed-up for a relatively long period of time. Limitations which need to be considered when interpreting the results of this study are the following: First, due to observational study design, we are not able to prove causality. Second, despite the liberal inclusion criteria, there is always a possibility of selection bias, or residual confounding. Third, the patient population consists mainly of white individuals and the generalizability of our results is unclear. Moreover, echocardiographic data was not systematically available. These data would have been useful to improve the characterization of the study population. Fourth, updating covariates such as AF type on a yearly basis may have induced some imprecision in the variable classification.

### Conclusion

In this large cohort of patients with AF, we found several potentially modifiable predictors of HF, providing potential opportunities for the implementation of preventive strategies. Risk factors were similar whether patients had known HF at baseline or not. Future studies should assess whether modifying these risk factors can decrease the risk for HF in AF patients.

## Supporting information

S1 Table Predictors of hospitalization for heart failure (HF) in patients with and without known HF at baseline by using subdistribution hazard models. (DOCX)

S1 File Questionnaire. (PDF)
