# Supplementary material for: Risk factors for heart failure hospitalizations among patients with atrial fibrillation
Source: PLoS One. 2018 Feb 2;13(2):e0191736. doi: 10.1371/journal.pone.0191736 (PMC5823079; doi:10.1371/journal.pone.0191736)
Supplement: S1 Table — (DOCX) [file pone.0191736.s001.docx]

Supplement

**Risk factors for heart failure hospitalizations among patients with atrial fibrillation**

Lucien Eggimann, Steffen Blum, Stefanie Aeschbacher, Andreas Reusser, Peter Ammann, Paul Erne, Giorgio Moschovitis, Marcello Di Valentino, Dipen Shah, Jürg Schläpfer, Nadine Mondet, Michael Kühne, Christian Sticherling, Stefan Osswald, David Conen

**Table S1:** Predictors of hospitalization for heart failure (HF) in patients with and without known HF at baseline by using subdistribution hazard models

|  | **HR (95% CI)** | **p-value** |
| --- | --- | --- |
| **Predictor in patients with and without known HF at baseline** | **n=1193** |  |
| BMI (kg/m^2^) | 1.38 (1.14; 1.67) | 0.0008 |
| Diastolic blood pressure (mmHg) | 0.85 (0.69; 1.05) | 0.114 |
| Chronic kidney disease | 2.36 (1.49; 3.44) | 0.0001 |
| Diabetes mellitus | 1.99 (1.30; 3.03) | 0.001 |
| History of arrhythmia intervention | 0.62 (0.41; 0.93) | 0.021 |
| QTc interval (ms) | 1.18 (0.95; 1.45) | 0.132 |
| BNP (ng/L) | 1.97 (1.55; 2.51) | <0.0001 |
| Chloride (mmol/l) | 0.81 (0.70; 0.95) | 0.009 |
| **Predictor in patients without known HF at baseline** | **n=951** |  |
| Age (years) | 1.42 (0.93; 2.17) | 0.102 |
| BMI (kg/m^2^) | 1.49 (1.15; 1.92) | 0.003 |
| Diabetes mellitus | 2.60 (1.51; 4.48) | 0.0006 |
| History of valve surgery | 3.08 (1.60; 5.95) | 0.0008 |
| History of arrhythmia intervention | 0.45 (0.24; 0.83) | 0.011 |
| QTc interval (ms) | 1.40 (1.07; 1.83) | 0.015 |
| BNP (ng/L) | 1.78 (1.18; 2.68) | 0.006 |

Data are Hazard Ratios (HR) and 95% confidence intervals (95%CI). HR for continuous
variables are per one standard deviation increase. BMI=body mass index; BNP=brain
natriuretic peptide; history of arrhythmia intervention =previous pulmonary vein ablation or/and
electrical cardioversion. BNP was log-transformed.
