# Supplementary material for: Risk factors for heart failure hospitalizations among patients with atrial fibrillation
Source: PLoS One. 2018 Feb 2;13(2):e0191736. doi: 10.1371/journal.pone.0191736 (PMC5823079; doi:10.1371/journal.pone.0191736)
Supplement: S1 File — (PDF) [file pone.0191736.s002.pdf]

38.00 Medical history

38.01 When was the patient's first detected atrial fibrillation? ☐☐ . ☐☐☐☐ (m m . j j j j)

38.02 How was atrial fibrillation documented? ***(Documentation requested!)***

☐ ***12-lead ECG*** ☐ ***Holter ECG*** ☐ ***Loop recorder / R-Test*** ☐ ***PM / ICD*** ☐ ***Other***

38.03 Current atrial fibrillation type: ☐ ***paroxysmal*** ☐ ***persistent*** (>7 days, ECV) ☐ ***permanent***

38.04 How often does the patient have episodes of atrial fibrillation? (Please only mark one answer)

☐ ***> 1 x per week*** ☐ ***< 1 x per week but > 1 x per month*** ☐ ***< 1 x per month*** ☐ ***no more episodes***

38.05 Mean duration of the episodes? ☐ ***minutes*** ☐ ***hours*** ☐ ***days*** ☐ ***no more episodes***

38.06 Which symptoms related with atrial fibrillation does the patient present?

☐ Palpitations ☐ Dizziness ☐ Chest pain ☐ none

☐ Dyspnea ☐ Fatigue ☐ Syncope ☐ Others ______

38.07 Are there any factors / behaviors that trigger atrial fibrillation in the patient?

☐ no ☐ Alcool ☐ Coffein ☐ Stress ☐ Lack of sleep/Tiredness

Others ______

38.08 Known atrial flutter? ☐ *Yes* ☐ *No*

38.09 H/o. RFA Isthmus? ☐ *Yes* ☐ *No* ***If so, when:*** ☐☐ . ☐☐☐☐ (m m . j j j j)

38.10 H/o. electrical cardioversion? ☐ *Yes* ☐ *No* ***If so:*** number ☐☐

38.11 H/o. PVI? ☐ *Yes* ☐ *No* ***If so:*** number ☐☐

38.12 H/o. Device implantation? ☐ *Yes* ☐ *No*

If so, Device type: ☐ ***PM*** ☐ ***ICD*** ☐ ***CRT*** ☐ ***CRT-ICD*** ☐ ***Loop recorder***

Date of implantation: ☐☐ . ☐☐ . ☐☐☐☐ (d d . m m . j j j j)

38.13 Procedural complications ? ☐ *Yes* ☐ *No* ***If so, which:*** ______

39.00 Anticoagulation/Medication

						morning	noon	evening
39.11	***Aspirin***	☐ *Yes*	☐ *No*	*Dosis*	______	☐	☐	☐
39.12	***OAC***	☐ *Yes*	☐ *No*	*If so, which:*	______	☐	☐	☐
39.13	***Anti-platelet medication***	☐ *Yes*	☐ *No*	*If so, which:*	______	☐	☐	☐
39.14	***Medication 1***	*Name:*	______	*Dosis*	______	☐	☐	☐
39.15	***Medication 2***	*Name:*	______	*Dosis*	______	☐	☐	☐
39.16	***Medication 3***	*Name:*	______	*Dosis*	______	☐	☐	☐
39.17	***Medication 4***	*Name:*	______	*Dosis*	______	☐	☐	☐
39.18	***Medication 5***	*Name:*	______	*Dosis*	______	☐	☐	☐
39.19	***Medication 6***	*Name:*	______	*Dosis*	______	☐	☐	☐

39.20 Which antiarrhythmic medication has the patient already been prescribed?

☐ none ☐ Flecainide ☐ Propafenon ☐ Sotalol ☐ Amiodaron ☐ Dronedaron ☐ others

When was the medication stopped? ☐☐☐☐ Reason for discontinuation: ______

39.30 ***Regular drug consumption?*** ☐ *Yes* ☐ *No*

If so, what kind of drug: ☐ ***Cocaine*** ☐ ***Heroin*** ☐ ***Marijuana*** ☐ ***Ecstasy*** ☐ ***LSD*** ☐ ***others***

40.00 Diseases

40.01	H/o. myocardial infarction	☐ Yes	☐ No		
40.02	H/o. CAB	☐ Yes	☐ No		
40.03	H/o. valve surgery	☐ Yes	☐ No	***Which:***	______
40.04	H/o. PCI / Stent	☐ Yes	☐ No		
40.05	H/o. Stroke / TIA	☐ Yes	☐ No		
40.06	Other embolism/vascular occlusion	☐ Yes	☐ No		
40.07	Known heart failure	☐ Yes	☐ No		
40.08	Obstructive sleep apnea syndrome	☐ Yes	☐ No		
40.09	Hypertension	☐ Yes	☐ No		
40.10	Diabetes mellitus	☐ Yes	☐ No		
40.11	Peripheral artery occlusive disease	☐ Yes	☐ No		
40.12	Renal failure	☐ Yes	☐ No		
40.13	Hyperthyroidism	☐ Yes	☐ No		
40.14	Hypothyroidism	☐ Yes	☐ No		
40.15	H/o. pulmonary embolism/DVT	☐ Yes	☐ No		
40.16	Known malignancy	☐ Yes	☐ No	***If so, organ:***	______
40.17	H/o. major bleeding	☐ Yes	☐ No		

If so: ☐ ***intracranial*** ☐ ***gastrointestinal*** ☐ ***other, requiring of blood transfusion*** ☐ ***others***

40.18	Gastric ulcer	☐ Yes	☐ No		
40.19	Recurrent falls	☐ Yes	☐ No		
40.20	Other serious diseases<br>***(e.g. gastrointestinal, nervous system, liver, etc.)***	☐ Yes	☐ No	***If so, which:***	______

41.00 Proportion of INR values in the target range ☐☐ / ☐☐ (over ☐☐ weeks)

42.00 Family history

42.10 Does a member of the patient's family suffer from atrial fibrillation?

42.11	***Father atrial fibrillation***	☐ *unknown*	☐ *No*	☐ *Yes*	***diagnosed at the age of:*** ☐☐	***years***
42.12	***Brother atrial fibrillation***	☐ *unknown*	☐ *No*	☐ *Yes*	***diagnosed at the age of:*** ☐☐	***years***
42.13	***Mother atrial fibrillation***	☐ *unknown*	☐ *No*	☐ *Yes*	***diagnosed at the age of:*** ☐☐	***years***
42.14	***Sister atrial fibrillation***	☐ *unknown*	☐ *No*	☐ *Yes*	***diagnosed at the age of:*** ☐☐	***years***

42.20 Is there a known family burden for the following diseases?

42.21	***Hypertension***	☐ *unknown*	☐ *No*	☐ *Yes*
42.22	***Diabetes mellitus***	☐ *unknown*	☐ *No*	☐ *Yes*
42.23	***Overweight***	☐ *unknown*	☐ *No*	☐ *Yes*
42.24	***CAD***	☐ *unknown*	☐ *No*	☐ *Yes*

43.00 Physical examination

43.10	Peripheral edema	☐ ***Yes***	☐ ***No***
43.20	Rales	☐ ***Yes***	☐ ***No***

44.00 Preliminary examinations *[Check-List]*

44.10	ECG done	☐	***Date:***	☐☐ . ☐☐ . ☐☐☐☐ (*d d . m m . j j j j*)
44.20	Holter-ECG done	☐	***Date:***	☐☐ . ☐☐ . ☐☐☐☐ (*d d . m m . j j j j*)
44.30	Echocardiography done	☐	***Date:***	☐☐ . ☐☐ . ☐☐☐☐ (*d d . m m . j j j j*)
44.40	Blood sampling	☐	***Date:***	☐☐ . ☐☐ . ☐☐☐☐ (*d d . m m . j j j j*)

45.00 Vital signs

45.10	Height	☐☐☐	***cm***
45.20	Body weight	☐☐☐	***kilograms***
45.30	Heart rate [manually over 30 seconds]	☐☐☐	***bpm***
45.40	Heart rate [ECG]	☐☐☐	***bpm***
45.50	Blood pressure - systolic, laid down [1]	☐☐☐	***mmHg***
45.60	Blood pressure - diastolic, laid down [1]	☐☐☐	***mmHg***
45.70	Blood pressure - systolic, laid down [2]	☐☐☐	***mmHg***
45.80	Blood pressure - diastolic, laid down [2]	☐☐☐	***mmHg***

46.00 Remarks
